# Pelvic osteotomies for acetabular dysplasia: development and current concepts in Japan

**DOI:** 10.1007/s00264-026-06980-3

**Published:** 2026-08-12

**Authors:** Takeyuki Tanaka, Toru Moro, Sakae Tanaka

**Affiliations:** 1 Orthopaedic Surgery, Sensory and Motor System Medicine, Surgical Sciences, Graduate School of Medicine, The University of Tokyo, Tokyo, Japan; 2 Division of Musculoskeletal AI System Dvelopment, Graduate School of Medicine, The University of Tokyo, Tokyo, Japan; 3 Division of Science for Joint Reconstruction, Graduate School of Medicine, The University of Tokyo, Tokyo, Japan

**Keywords:** Acetabular dysplasia, Developmental dysplasia of the hip, Pelvic osteotomy, Joint-preserving surgery, Clinical outcome

## Abstract

Acetabular dysplasia is a leading cause of secondary hip osteoarthritis in Japan and has long been a major indication for joint-preserving surgery. Over the past decades, several unique pelvic osteotomy techniques have been developed in Japan. Representative procedures include Transposition Osteotomy of the Acetabulum, Rotational Acetabular Osteotomy, Eccentric Rotational Acetabular Osteotomy, Curved Periacetabular Osteotomy, and Spherical Periacetabular Osteotomy. Although these procedures share the common objective of improving femoral head coverage and preserving the native hip joint, each procedure has evolved independently with distinct surgical concepts, osteotomy designs, and technical characteristics. Numerous studies have demonstrated favourable clinical outcomes after these procedures when appropriate patient selection is performed. Recent advances in three-dimensional imaging, computer simulations, and computer-assisted surgery have further refined surgical planning and postoperative assessments. This review summarises the historical evolution, surgical concepts, indications, clinical outcomes, and future perspectives of pelvic osteotomies developed in Japan, highlighting their individual characteristics and continuing roles in contemporary joint-preserving hip surgeries.

## Introduction

Osteoarthritis (OA) of the hip is a debilitating disease that causes pain, restricted range of motion, limb-length discrepancy, muscle weakness, and gait disturbance, substantially impairing the activities of daily living and quality of life. Various risk factors for hip OA have been reported, including occupations involving heavy physical labour or prolonged standing, obesity, participation in high-impact sports, acetabular dysplasia, and a history of developmental dysplasia (or dislocation) of the hip [1–5]. Among these, acetabular dysplasia is by far the most common cause of secondary hip OA in Japan, accounting for more than 70% of affected hips, whereas primary OA is considerably more prevalent in Western countries [6]. This difference in patient backgrounds has greatly influenced the evolution of joint-preserving hip surgeries in Japan.

Several radiographic parameters have been used to evaluate acetabular dysplasia, including lateral centre-edge (CE) angle, sharp angle, acetabular roof obliquity (ARO), and acetabular head index (AHI). Among these, CE angle remains the most widely accepted parameter for assessing the severity of dysplasia, determining surgical indications, and evaluating postoperative outcomes. Ethnic differences in acetabular morphology have also been reported, with Japanese individuals, particularly women, demonstrating smaller CE angles than Western populations [7]. These anatomical characteristics provide the basis for the development of various joint-preserving pelvic osteotomies in Japan.

Several unique pelvic osteotomy procedures have been developed in Japan, including the Transposition Osteotomy of the Acetabulum (TOA) [8, 9], Rotational Acetabular Osteotomy (RAO) [10–12], Eccentric Rotational Acetabular Osteotomy (ERAO) [13, 14], Curved Periacetabular Osteotomy (CPO) [15, 16], and Spherical Periacetabular Osteotomy (SPO) [17, 18]. Although these procedures share the common principle of spherical acetabular reorientation, they differ considerably from the Bernese periacetabular osteotomy described by Ganz [19], which is based on multiplanar osteotomies around the acetabulum. Furthermore, each Japanese procedure has evolved independently and possesses distinct surgical concepts, osteotomy designs, and approaches.

One of the principal differences among these procedures is whether osteotomies of the quadrilateral surface (QLS) and pubis are performed. Historically, most Japanese pelvic osteotomies have been performed using the lateral approach, with the patient in the lateral decubitus position. More recently, however, several procedures utilising a single anterior approach in the supine position have been developed. Another important aspect is that the surgical techniques of TOA and RAO have evolved considerably since their original descriptions. While the original TOA included osteotomies of the QLS and pubis and the original RAO did not, subsequent technical modifications have narrowed these differences, and contemporary techniques often include osteotomies of both structures. In contrast, ERAO has consistently incorporated osteotomies of the QLS and pubis while modifying the centre of rotation to minimise the proximal displacement of the femoral head. The major difference between the above-mentioned three procedures and the more recently developed CPO and SPO is the surgical approach, which is performed entirely through a single anterior approach with the patient in the supine position, whereas the principal difference between CPO and SPO is whether osteotomies of the QLS and pubis are performed.

The purpose of this review is to summarize the historical development, current concepts, surgical characteristics, clinical outcomes, and future perspectives of pelvic osteotomies developed in Japan for the treatment of acetabular dysplasia.

### Patient selection and surgical indications

Appropriate patient selection is the cornerstone of a successful pelvic osteotomy, and general indications are largely shared among the currently available procedures developed in Japan [20]. Ideal candidates are symptomatic patients with acetabular dysplasia, pre-osteoarthritis, or early stage osteoarthritis. In patients with early advanced osteoarthritis, preservation of adequate joint space remains essential. Hasegawa et al. recommended that a minimum joint space width of 2mm should be maintained when considering joint-preserving surgery [21].

Joint congruity is another critical factor that influences surgical indications. Excellent or good congruity on abduction anteroposterior radiographs is generally regarded as a prerequisite for successful pelvic osteotomy, because satisfactory congruity after acetabular reorientation is closely associated with favourable long-term outcomes [22].

Although the precise indications differ slightly among procedures and institutions, the lateral CE angle typically ranges from approximately -30° to 10°. Pelvic osteotomy is generally indicated after the closure of the triradiate cartilage, usually from approximately 12 to the fifth decade of life. Therefore, careful evaluation of patient age, osteoarthritic stage, joint congruity, and radiographic severity of acetabular dysplasia is essential for selecting optimal candidates for joint-preserving surgery.

### Transposition Osteotomy of the Acetabulum (TOA)

Transposition Osteotomy of the Acetabulum (TOA) is regarded as the earliest pelvic osteotomy developed for the treatment of acetabular dysplasia and can be traced back to the procedure first described by Nishio in 1956 [8]. Although the original report was published in Japanese, it was introduced to the international community several decades later [9]. Since its introduction, the surgical technique has undergone several modifications in terms of osteotomy design and surgical approach.

Contemporary TOA is generally performed with the patient in the lateral decubitus position and usually includes osteotomies of both the QLS and pubis. One of the most important technical developments is the muscle-sparing approach proposed by Nakashima et al. [23]. Instead of conventional greater trochanteric osteotomy, osteotomies are performed through the anterior and posterior intervals of the gluteus medius and minimus muscles, thereby minimising damage to the hip abductor musculature while preserving adequate surgical exposure. This modification has contributed to the reduction of postoperative abductor dysfunction without compromising surgical accuracy.

Structural bone grafting is not routinely required during TOA, although surgical techniques vary among institutions. Potential complications include fractures around the osteotomy site and vascular injury during acetabular mobilisation. Despite these concerns, TOA has demonstrated favourable long-term clinical outcomes and remains an important joint-preserving procedure in Japan.

### Rotational Acetabular Osteotomy (RAO)

**Fig. 1:**
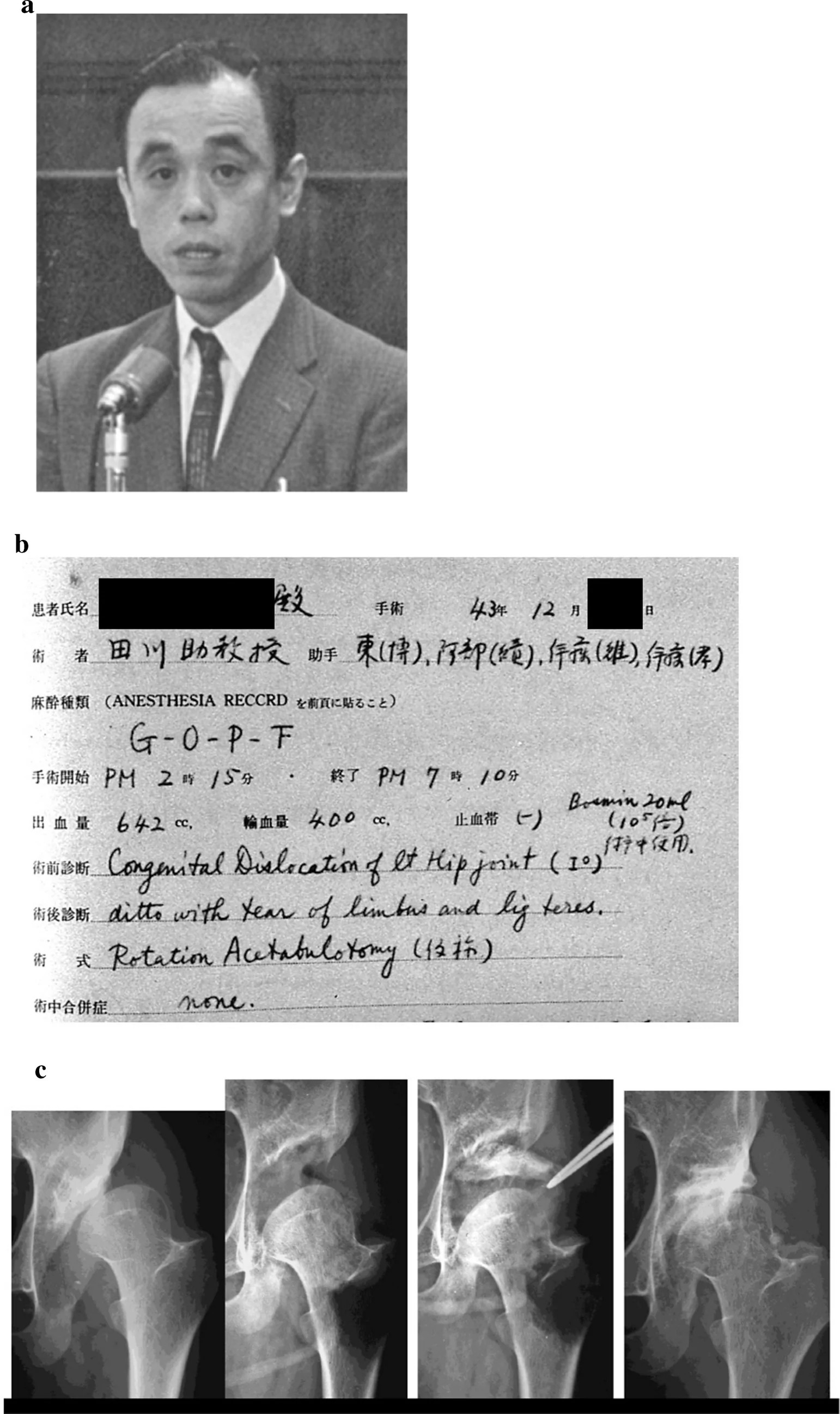
**a** Dr. Hiroshi Tagawa, who established rotational acetabular osteotomy (RAO). **b** Original operative record of the first RAO performed in 1968. **c** Serial anteroposterior radiographs of the first patient treated with RAO: preoperative, intraoperative, immediate postoperative, and 4 months postoperatively

Rotational Acetabular Osteotomy (RAO) is one of the best known pelvic osteotomy procedures developed in Japan. The concept originated from the neoacetabuloplasty described by Iino in 1968 [24], and the surgical technique was subsequently established by Tagawa (Fig. 1a–c). The original Japanese report was published in 1974 [10], followed by the first English-language publication by Ninomiya and Tagawa in 1984 [11], through which the RAO became internationally recognised.

**Fig. 2:**
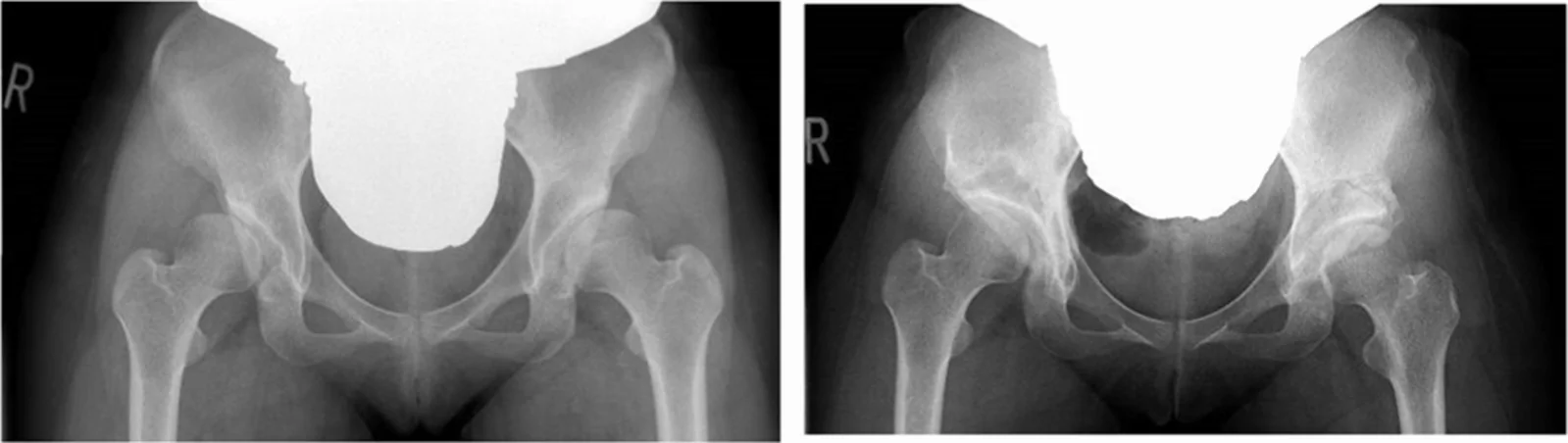
Preoperative and postoperative radiographs of a representative bilateral RAO case. The postoperative radiograph of the right hip was obtained 3 years after surgery, demonstrating complete union of the osteotomy site. The postoperative radiograph of the left hip was obtained 3 months after surgery, when the osteotomy line remains visible before complete union. Modified from Tanaka T et al. [45] under the terms of the Creative Commons Attribution 4.0 International License (CC BY 4.0)

The original RAO was characterised by a spherical acetabular osteotomy without osteotomy of the QLS or pubis. However, in the mid-1980s, Ninomiya introduced several important modifications, including a thicker acetabular fragment and additional osteotomies of the QLS and pubis [25]. These modifications improved fragment stability and facilitated acetabular reorientation while avoiding intra-articular osteotomies. Consequently, contemporary RAO differs substantially from the original procedure, and has become the standard technique adopted by many institutions in Japan (Fig. 2).

**Fig. 3:**
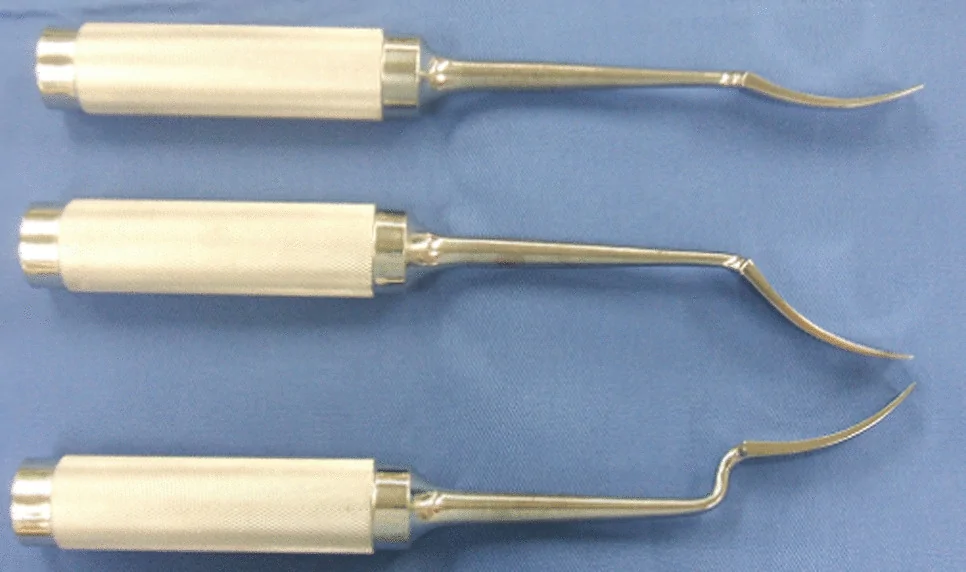
Specially designed curved osteotomes

One of the characteristic features of RAO is the use of a specially designed curved osteotome that enables spherical osteotomy around the acetabulum (Fig. 3). Curved osteotomes are commonly used in almost Japanese pelvic osteotomies. Greater trochanteric osteotomy may or may not be performed depending on the surgeon’s preference; however, transient postoperative weakness of the hip abductors may occur regardless of the surgical approach. Structural autograft grafting is commonly performed to fill the gap between the rotated acetabular fragment and the remaining pelvis. Several institutions have recently adopted artificial bone substitutes.

Although RAO demonstrates excellent long-term survival, it is technically demanding. The reported complications include fractures around the osteotomy site and bleeding associated with pubic osteotomy. Nevertheless, RAO remains one of the most extensively investigated pelvic osteotomy procedures worldwide, and serves as the foundation for several subsequent modifications developed in Japan.

### Eccentric Rotational Acetabular Osteotomy (ERAO)

Eccentric Rotational Acetabular Osteotomy (ERAO) was developed by Hasegawa. The first original Japanese report was published in 1998 [13] as a modification of the RAO and was first introduced internationally in 2002 [14]. The procedure is performed in the lateral decubitus position and has consistently incorporated a greater trochanteric osteotomy since its original description. This approach provides excellent surgical exposure, facilitates safe osteotomy, and secures fixation of the rotated acetabular fragment. Improved visualisation has also been associated with reduced postoperative pain and earlier functional recovery.

The principal characteristic of ERAO is its modified centre of rotation. Compared with conventional TOA or RAO, the centre of spherical osteotomy is shifted medially and superiorly, allowing medialization and distalisation of the femoral head after acetabular reorientation. Consequently, structural bone grafting, which is frequently required in conventional RAO, is generally unnecessary in ERAO. ERAO usually includes osteotomies of both the QLS and the pubis. This biomechanical concept is one of the major differences between the two procedures.

Potential disadvantages include temporary abductor weakness related to greater trochanteric osteotomy, and delayed union or nonunion of the pubic osteotomy site. Nevertheless, long-term clinical studies have demonstrated favourable survivorship, establishing ERAO as a reliable alternative to conventional TOA or RAO in appropriately selected patients.

### Curved Periacetabular Osteotomy (CPO)

Curved Periacetabular Osteotomy (CPO) was developed by Naito, and the first original Japanese report was published in 1998 [15]. The first English language report was published in 2005 [16]. Although this concept was derived from the Bernese periacetabular osteotomy described by Ganz, CPO differs fundamentally in that acetabular reorientation is achieved by a spherical osteotomy rather than multiple straight osteotomies. Since its introduction, CPO has become a representative anterior approach pelvic osteotomy procedure performed in Japan.

Unlike conventional TOA, RAO, and ERAO, CPO is performed entirely through a single anterior approach, with the patient in the supine position. Osteotomies of both the quadrilateral surface and pubis were routinely performed. Compared with lateral-approach procedures, CPO offers several potential advantages, including a smaller skin incision, preservation of the hip abductor muscles, and improved visualisation of the pubic osteotomy site. These characteristics have contributed to its increasing popularity, particularly among surgeons who prefer the anterior approach.

Structural bone grafting is generally unnecessary, because the acetabular fragment can be repositioned without creating substantial osseous defects. Reported complications include lateral femoral cutaneous nerve palsy and delayed union of the pubic osteotomy site. Despite these potential limitations, CPO has demonstrated favourable short- and mid-term clinical outcomes.

### Spherical Periacetabular Osteotomy (SPO)

**Fig. 4:**
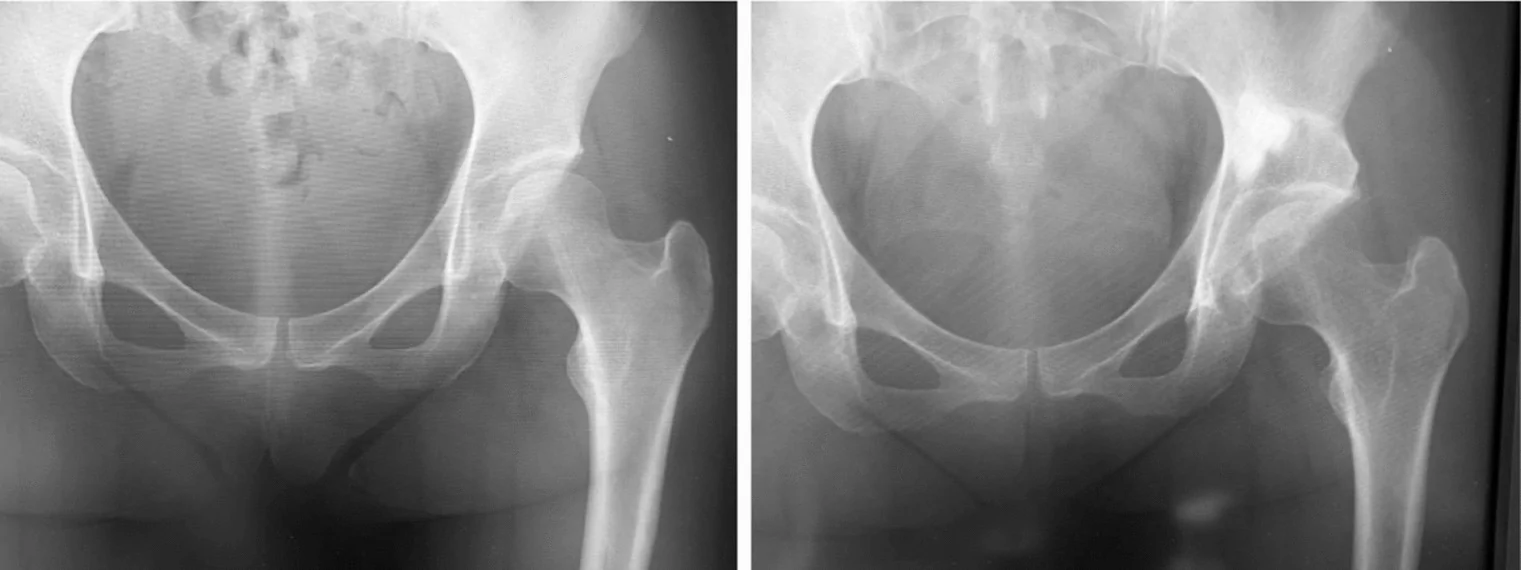
Preoperative and postoperative radiographs of a representative spherical periacetabular osteotomy (SPO) case. Unlike other procedures, osteotomies of the quadrilateral surface and pubis were not performed. The osteotomy gap was filled with a β-tricalcium phosphate bone substitute. Representative radiographs provided by Dr. Toru Nishiwaki with permission

Spherical Periacetabular Osteotomy (SPO) was first reported by Hara in 2004 [17] and later reported internationally by Kaneuji et al. in 2021 [18]. Similar to the CPO, the procedure was performed using a single anterior approach with the patient in the supine position. However, unlike the CPO, the SPO does not require osteotomies of either the quadrilateral surface or the pubis. Instead, the osteotomy line resembled that of the original RAO using a minimally invasive anterior approach (Fig. 4).

This procedure has several advantages. Preservation of the QLS prevents disruption of the pelvic floor and may reduce concerns regarding the birth canal in female patients. Smaller skin incision and the preservation of pelvic continuity also contribute to its minimally invasive nature. In many cases, β-tricalcium phosphate or other artificial bone substitutes are inserted into the osteotomy gap instead of autologous bone grafts.

The potential disadvantages include lateral femoral cutaneous nerve palsy and postoperative limb shortening. Furthermore, because the osteotome is advanced between the radiographic teardrops under fluoroscopic guidance, inadvertent penetration of the hip joint may occur in selected cases.

The characteristic features of currently available pelvic osteotomies are summarised in Table 1. As these procedures have evolved considerably since their original descriptions, the table summarises the representative characteristics of contemporary techniques rather than providing definitive distinctions between procedures.

**Table 1:** Representative characteristics of contemporary Japanese pelvic osteotomy procedures

Procedure	Patient position	Greater Trochanteric osteotomy	QLS and pubis osteotomies	Bone grafting
TOA	Lateral Decubitus	Sometimes	Usually	Sometimes
RAO	Lateral Decubitus	Sometimes	Usually	Usually
ERAO	Lateral Decubitus	Always	Usually	Unnecessary
CPO	Supine	None	Usually	Sometimes
SPO	Supine	None	none	Usually

Abbreviations: *TOA* Transposition Osteotomy of the acetabulum, *RAO* Rotational Acetabular osteotomy, *ERAO* Eccentric Rotational Acetabular Osteotomy, *CPO* Curved Periacetabular Osteotomy, *SPO* Spherical Periacetabular Osteotomy

### Clinical outcomes

Clinical outcomes are among the most important factors in evaluating the effectiveness of pelvic osteotomy. Representative long-term studies on Japanese pelvic osteotomies are summarised in Table 2. Although direct comparison among procedures remains difficult because of differences in patient characteristics, disease severity, follow-up duration, and study endpoints, the overall clinical outcomes are generally favourable across all procedures when appropriate patient selection is applied.

**Table 2:** Summary of representative clinical outcome studies of Japanese pelvic osteotomy procedures

Author	Procedure	No. of Hips	Mean age at surgery	Mean follow-up after surgery	Survival rate (%) Endpoint: OA progression	Survival rate (%) Endpoint: THA conversion	Postoperative CROs	Postoperative PROs
Karashima et al. (2011)	CPO	191	MD: 36.5 SD: 36.4	MD: 70.9 months SD: 70.6 months	Overall: 87.4	MD: 98.0 SD: 100.0	HHS MD: 94.3 SD: 94.7	NA
Hasegawa et al. (2014)	ERAO	130	37.4 (15–59)	19.7 (15–23) years	NA	20-year KM Overall: 87.3 Pre OA: 97.4 Early OA: 79.6 Advanced OA:73.8	HHS 80 (35–100)	NA
Kaneuji et al. (2015)	RAO	93	32.4 (12–49)	Pre OA: 22.5 years Early OA: 22.8 years Advanced OA: 20.0 years	Pre OA: 87.0 Early OA:89.7 Advanced OA: 53.7	Pre OA: 73.9 Early OA: 65.5 Advanced OA: 63.4	HHS Pre OA: 89.4 Early OA: 84.5 Advanced OA: 84.1	JHEQ (Overall) Pre OA: 69.7 Early OA: 55.5 Advanced: 54.6
Yuasa et al. (2017)	RAO	115	41.4 (14–60)	20 years and 4 months (15–26 years and 7 months)	Pre OA: 66.7 Early OA: 63.2 Advanced OA: 55.9	20-year KM Pre OA: 88.9, Early OA: 78.9 Advanced OA: 59.3	HHS Pre OA: 83.3 Early OA: 87.4 Advanced OA: 71.2	NA
Kaneuji et al. (2021)	SPO	55	38.0 (17–56)	37.4 (24.0–61.2) months	98.2	100.0	HHS 89.6	JHEQ (Overall) 63.5
Nakashima et al. (2022)	TOA	172	42.9 (13.5–64.3)	21.0 (16.6–24.6) years	NA	20-year KM Overall: 79.7 Pre OA: 93.3 Early OA: 86.7 Advanced OA: 54.8	NA	VAS Satisfaction: 81.5 VAS Pain 17.7 OHS 42.4 FJS-12 59.2
Yasunaga et al. (2024)	RAO	43	Pre OA: 23.8 (13–51) Early OA: 37.3 (22–58)	Minimum of 28 years	30-year KM Overall: 51.5 Pre OA: 81.8 Early OA: 42.2	30-year KM Overall: 74.0	Merle d'Aubigné score Pre OA 17.4 Initial OA 14.6	NA
Kitamura et al. (2026)	TOA	392	BD: 42.5 FD: 42.4	BD: 14.5 FD: 13.9	NA	20-year KM BD: 90.0 FD: 78.8	NA	BD: VAS Satisfaction: 91 VAS Pain 10 OHS 45 FJS-12 60 FD: VAS Satisfaction: 91 VAS Pain 10 OHS 43 FJS-12 54

Abbreviations: *TOA* Transposition Osteotomy of the acetabulum, *RAO* Rotational Acetabular osteotomy, *ERAO* Eccentric Rotational Acetabular Osteotomy, *CPO* Curved Periacetabular Osteotomy, *SPO* Spherical Periacetabular Osteotomy, *OA* osteoarthritis, *THA* Total Hip Arthroplasty, *CROs* Clinician-reported Outcomes, *PROs* Patient-reported Outcomes, *MD* Moderate Dysplasia, *SD* Severe Dysplasia, *HHS* Harris Hip Score, *NA* not available, *KM* Kaplan–Meier, *VAS* visual analogue scale, *OHS* Oxford hip score, *FJS* Forgotten joint score, *JHEQ* Japanese Orthopaedic Association hip-disease evaluation questionnaire, *BD* borderline dysplasia, *FD* frank dysplasia

### Transposition Osteotomy of the Acetabulum (TOA)

As a pioneering pelvic osteotomy technique developed in Japan, TOA has demonstrated favourable long-term clinical outcomes. Nakashima et al. reported significant improvements in patient-reported outcome measures after a mean follow-up period of 21years in 172 hips [26]. The 20-year Kaplan–Meier survivorship, with conversion to total hip arthroplasty (THA) as the endpoint, was 93.3% for pre-osteoarthritis, 86.7% for early stage osteoarthritis, and 54.8% for advanced-stage osteoarthritis.

More recently, Kitamura et al. compared the long-term outcomes of frank hip dysplasia (lateral CE angle < 18°) and borderline hip dysplasia (lateral CE angle 18–25°) [27]. The 20-year Kaplan–Meier survivorship rate, with conversion to THA as the endpoint, was 78.8% in patients with frank dysplasia and 90.0% in those with borderline dysplasia, suggesting that favourable long-term outcomes can also be achieved in carefully selected patients with acetabular dysplasia.

### Rotational Acetabular Osteotomy (RAO)

Among the Japanese pelvic osteotomies, RAO has accumulated the largest body of long-term clinical evidence.

Kaneuji et al.. evaluated 93 hips (23 pre-OA, 29 early-OA, and 41 advanced-OA) after a mean follow-up period of 23 years [28]. Hip survivorship, with radiographic progression of OA as the endpoint, was 73.9% for pre-OA, 65.5% for early-OA, and 63.4% for advanced-OA. The hip survivorship rates with conversion to THA as the endpoint were 87.0%, 89.7%, and 46.3%, respectively.

Yuasa et al. reported the long-term results of 115 hips (18 pre-OA, 38 early-OA and 59 advanced-OA) after a mean follow-up of 20.4 years [29]. Hip survival, with radiographic progression as the endpoint, was 66.7%, 63.2%, and 55.9%, respectively. The 20-year Kaplan–Meier survivorship, with conversion to THA as the endpoint, was 88.9% for pre-OA, 78.9% for early-OA, and 59.3% for advanced-OA.

Yasunaga et al. reported the longest follow-up to date, evaluating 43 hips (11 pre-OA and 32 early-OA) with a minimum follow-up of 28 years [30]. Hip survivorship, with radiographic progression as the endpoint, was 81.8% in pre-OA patients and 56.3% in early-OA. The 30-year Kaplan–Meier survivorship rates were 81.8% and 42.2%, respectively, with conversion to THA as the endpoint. Overall 30-year Kaplan–Meier survival rate was 51.5% with radiographic progression as the endpoint and 74.0% with conversion to THA as the endpoint.

Collectively, these studies demonstrated favourable long-term outcomes after RAO, particularly in patients with pre-and early osteoarthritis, emphasising the importance of appropriate patient selection and timely surgical interventions.

### Eccentric Rotational Acetabular Osteotomy (ERAO)

Hasegawa et al. reported the long-term outcomes of 130 hips after eccentric rotational acetabular osteotomy (ERAO) in patients with a mean age of 37 years after a mean follow-up of 20 years [31]. The 20-year Kaplan–Meier survivorship rate, with conversion to THA as the endpoint, was 87.3%. According to disease stage, the 20-year Kaplan–Meier survivorship was 97.4% for pre-OA, 79.6% for early-OA, and 73.8% for advanced-OA, including hips treated with concomitant femoral valgus osteotomy.

### Curved Periacetabular Osteotomy (CPO) and Spherical Periacetabular Osteotomy (SPO)

Long-term follow-up studies on curved periacetabular osteotomy (CPO) and spherical periacetabular osteotomy (SPO) are limited because these procedures were introduced after the lateral decubitus osteotomies described above. However, favourable short- to mid-term clinical outcomes have been reported.

Karashima et al. evaluated 191 hips undergoing CPO, including 147 hips with moderate dysplasia (lateral CE angle ≥ 0°) and 44 hips with severe dysplasia (lateral CE angle < 0°), after a minimum follow-up of two years (mean follow-up, 70.9 months and 70.6 months, respectively) [32]. At the final follow-up, 87.4% of the hips maintained their preoperative osteoarthritis stage, and only three hips, all in the moderate dysplasia group, required conversion to THA.

Kaneuji et al. reported the short-term outcomes of 55 hips treated with SPO (mean age, 38 years), including 27 pre-OA, 21 early-OA, and seven advanced-OA hips, after a mean follow-up of 37.4 months [18]. Radiographic progression was observed in only one pre-osteoarthritic hip, and none of the patients required conversion to THA during follow-up.

### Future perspectives

Traditionally, surgical indications for pelvic osteotomy and postoperative outcome assessment have been primarily based on two-dimensional radiographic parameters, including the lateral centre-edge (CE) angle. Although these radiographic measurements are useful in daily clinical practice, they do not always reflect the complex three-dimensional morphology of the acetabulum.

In clinical practice, some patients undergo pelvic osteotomy after careful selection and demonstrate satisfactory postoperative acetabular coverage on conventional anteroposterior radiographs, yet subsequently require conversion to THA within a relatively short period [33], although the reasons for this are likely multifactorial. These observations suggest that conventional two-dimensional evaluation alone may be insufficient for predicting long-term clinical outcomes and highlight the importance of three-dimensional assessment of acetabular morphology. Indeed, in recent years, three-dimensional analyses of various pelvic osteotomy procedures, including simulation studies, have been increasingly reported, further emphasising the clinical value of three-dimensional evaluation [34–39].

Recent advances in computed tomography, three-dimensional reconstruction, and computer simulations have substantially improved our understanding of the acetabular morphology before and after pelvic osteotomies. In particular, three-dimensional evaluations of anterior and posterior acetabular coverage, acetabular version, and overall coverage balance have provided insights that cannot be obtained using conventional radiographic parameters alone. Consequently, future surgical indications and postoperative assessments may increasingly incorporate three-dimensional parameters in addition to traditional radiographic measurements.

Another important issue is the preservation and transmission of surgical expertise. Owing to continuous improvements in total hip arthroplasty, including advances in surgical techniques and implant longevity, the indications for THA have expanded to increasingly younger patients [40]. Consequently, the number of pelvic osteotomies performed is expected to gradually decline in many institutions. Nevertheless, periacetabular osteotomy remains an indispensable joint-preserving procedure in appropriately selected young patients with acetabular dysplasia.

Pelvic osteotomy is technically demanding and requires precise osteotomy, accurate acetabular reorientation, and a thorough understanding of three-dimensional pelvic anatomy. Although the mastery of these techniques has traditionally depended on surgical experience, maintaining adequate opportunities for technical training may become increasingly challenging as the surgical case volume decreases. Therefore, technologies capable of accurately reproducing preoperative surgical plans are becoming increasingly valuable. Computer-assisted surgery (CAS), particularly computed tomography-based navigation systems, has the potential to improve surgical accuracy, enhance reproducibility, and facilitate surgical education by providing visual guidance during complex osteotomies.

Several authors have recently reported encouraging early experiences with navigation-assisted rotational acetabular osteotomies. Although long-term clinical evidence remains limited, continued technological advances and the accumulation of clinical data are expected to further establish the role of computer-assisted surgery in pelvic osteotomy [41–44].

## Conclusions

Pelvic osteotomy has evolved remarkably in Japan over the past several decades, resulting in the development of several unique surgical procedures for treating acetabular dysplasia.

Although TOA, RAO, ERAO, CPO, and SPO share the common objective of preserving the native hip joint, each procedure has distinct surgical concepts, technical characteristics, advantages, and limitations. A thorough understanding of these differences is essential for selecting the most appropriate procedure based on individual patient characteristics.

Long-term clinical studies have demonstrated favourable outcomes for most Japanese pelvic osteotomies, particularly when appropriate patient selection is applied. Recent advances in three-dimensional imaging, computer simulation, and computer-assisted surgery are expected to further improve surgical planning, operative accuracy, and postoperative assessments.

Japanese pelvic osteotomies continue to represent one of the most important contributions to joint-preserving hip surgeries worldwide. Continued refinement of surgical techniques, accumulation of long-term clinical evidence, and successful transmission of surgical expertise to future generations are essential for further development of this field.

## Data Availability

No datasets were generated or analysed during the current study.
